# Mpox in the Middle East and North Africa: Containment, Prevention, and Future Measures

**DOI:** 10.7759/cureus.87758

**Published:** 2025-07-11

**Authors:** Miriam Khair, Bilal Irfan

**Affiliations:** 1 Department of Global Health Research, Horizons Academy, East Jerusalem, PSE; 2 Center for Surgery and Public Health, Brigham and Women's Hospital, Boston, USA; 3 Michigan Alzheimer's Disease Center, University of Michigan, Ann Arbor, USA; 4 Center for Bioethics, Harvard Medical School, Boston, USA

**Keywords:** global health, infectious disease, middle east north africa region, mpox, regional response

## Abstract

Mpox has emerged as a global concern and warrants a global response. This editorial argues that the Middle East and North Africa region sits at a precarious intersection of strained health systems and porous borders. Clinical presentation is increasingly atypical, yet painful oral lesions and rash remain diagnostic anchors. Disproportionate burden among men, particularly those with intersecting immunocompromising conditions, underscores both behavioral and biological vulnerabilities. First-generation vaccinia stockpiles offer interim protection, but third-generation non-replicating vaccines, paired with strategic deployment, promise safer, longer-lasting immunity for genetically diverse communities. Real-time prevention capacity, transparent risk communication, regional antiviral mutual-aid pacts, and targeted vaccination of frontline workers and high-risk groups constitute a practical containment blueprint. Policymakers can avert an avoidable crisis by translating these evidence-based measures into pre-emptive action - before the next zoonotic roll of the dice - and by working in collaboration with other regional and international stakeholders.

## Editorial

Mpox, as named by the World Health Organization (WHO) in 2022, has re-emerged as a vivid reminder that the interval between zoonotic spillovers is shrinking. Five years after severe acute respiratory syndrome coronavirus 2 (SARS-CoV-2) upended health systems and economies alike, global vigilance remains understandably high, yet the capacity to act swiftly is unevenly distributed. That asymmetry is apparent in the Middle East and North Africa (MENA). Many states in this region have young populations, porous borders, and healthcare infrastructures strained by protracted conflict or fiscal austerity. For example, Jordan hosts a large number of refugees, which increases constraints on the healthcare sector's budget, and Lebanon has faced an exodus of many of its physicians. Those same features, which hampered the early COVID-19 response, could magnify the impact of any future mpox resurgence unless decisive containment and prevention measures are planned in advance.

Mpox was first detected in captive macaques shipped from Singapore in 1958. Although the name derives from that epizootic event, rodents - not non-human primates - are now believed to constitute the principal animal reservoir. Human infection went unrecognized until 1970, when a nine-month-old child in Zaire (now the Democratic Republic of the Congo) presented with a smallpox-like illness that proved, on viral isolation, to be mpox. Subsequent field investigations revealed sporadic zoonotic cases in equatorial Africa and, by 1997, documented limited chains of person-to-person transmission - an epidemiological turning point that points to persistent biosafety gaps across the region.

The global trajectory of the virus can be divided into three epochs. During the first, spanning 1970 to 2002, cases were geographically confined to central and western Africa. Beginning in 2003, air travel and exotic-pet commerce enabled exportations to the United States and Europe, inaugurating a second period of intercontinental spread. Then, in May 2022, a constellation of cases with no travel links to endemic zones signaled the opening of a third epoch: a true multinational outbreak that eventually exceeded 30,000 laboratory-confirmed infections within a single year [1]. Those three epochs are more than a historical footnote, as they illustrate how viral ecology, human mobility, and immunological landscape interact to modulate risk, and they inform the design of proportionate countermeasures for the MENA region.

Clinically speaking, mpox can masquerade as common dermato-viral illnesses, yet several features call for careful attention. Painful oral lesions, especially on the dorsal tongue, frequently precede or accompany the centrifugal rash. Although the prototypical lesion evolves from macule to papule to crust over two to three weeks, recent outbreaks have presented with fewer lesions, atypical anogenital distribution, and muted prodromal symptoms. While rash remains a cardinal sign across epochs, the prevalence of fever, lymphadenopathy, and myalgia has possibly declined, potentially reflecting altered inoculation sites, partial population immunity, or viral evolution [1].

Risk is not uniformly shared, and there are differences across gender and sexual activity. Social networks, health-seeking behavior, and surveillance bias partly explain that imbalance, yet biological susceptibility cannot be excluded. Intersectional vulnerabilities compound the picture. Immunocompromised individuals, whether because of advanced human immunodeficiency virus infection, solid-organ transplantation, or iatrogenic immunosuppression, may exhibit higher rates of hospitalization and death [2]. Although mpox case-fatality ratios have remained low by historical smallpox standards, even a modest uptick in virulence or a delay in access to care could prove devastating in settings where intensive-care capacity and antiviral stockpiles are limited.

Vaccination is, therefore, the linchpin of forward-looking preparedness [3]. First-generation vaccinia vaccines, long stockpiled under smallpox contingency plans, confer substantial cross-protection and are already approved for mpox. However, rare severe adverse events and contraindications in immunocompromised hosts diminish their population reach. Third-generation, non-replicating modified vaccinia Ankara (MVA) formulations elicit robust neutralizing antibodies with a superior safety profile. The challenge is not scientific feasibility, but equitable distribution and context-specific implementation. A bid to link host genomics, immunophenotype, and environmental modifiers can optimize vaccine selection, dosage, and scheduling for diverse ethnic groups. Such tailored approaches hold particular promise in MENA, where consanguinity rates, population admixture, and a breadth of haplotypes could influence vaccine take and durability of protection.

Yet, an efficacious vaccine is inert without public trust. Political instability, misinformation cascades, and religious concerns may all serve to erode confidence. In conflict-affected settings, for example, recurrent power cuts, damage to laboratory buildings, and episodes of looting or vandalism of healthcare infrastructure may leave the few remaining facilities operating with skeletal staff and unreliable supply pipelines. In different parts of the MENA region, where digital literacy varies widely and official communication channels may be viewed with skepticism, clinicians and public-health authorities must invest early in transparent engagement. Partnering with community leaders, faith-based organizations, and youth influencers can counter rumors and foster literacy. Parallel efforts to strengthen routine infection-prevention practices, prompt recognition of rash illness, meticulous hand hygiene, and mask usage in clinical triage areas may help to slow any incursion long enough for vaccines and antivirals to be deployed. Framing vaccine drives around core religious concepts of cleanliness, preventing harm, and promoting communal welfare, and delivered through Friday sermons or mosque-based health initiatives in Muslim-majority settings, may help in bridging barriers for vaccination efforts [4,5].

Laboratory capacity represents another line of defense. Polymerase chain-reaction (PCR) assays targeting some of the DNA are readily adaptable to hospital or national reference laboratories, yet the reagents, cold-chain logistics, and trained personnel required for reliable diagnostics are not uniformly available across MENA. Investments motivated by the coronavirus pandemic can be leveraged, such as modular biosafety cabinets, point-of-care PCR platforms, and open-source protocols to reduce cost barriers. Implementing biosafety recommendations to provide pragmatic guidance for upgrading facilities, in line with international norms, before - not during - an outbreak will prevent avoidable laboratory exposures and data bottlenecks.

A comprehensive containment strategy must also reckon with porous borders and complex humanitarian emergencies. Refugee flows, mass religious gatherings, and labor migration can accelerate cross-border transmission. The 2022 mpox outbreak reaffirmed that travel screening focused exclusively on travelers from historically endemic countries is insufficient. Instead, real-time syndromic surveillance, cross-jurisdictional data sharing, and pre-arranged antiviral mutual-aid agreements offer more resilient protection. Countries hosting large displaced populations should prioritize vaccination for front-line health workers and immunocompromised residents, recognizing that high-density shelters and limited sanitation amplify risk.

Although mpox has never matched the transmissibility of measles or the lethality of smallpox, complacency would be ill-advised. Genetic drift or recombination in a chronically infected, immunosuppressed individual could theoretically yield variants with enhanced transmissibility or immune escape. The low baseline seroprevalence in individuals born after the cessation of routine smallpox vaccination further widens the immunological niche. Eradication, therefore, remains the aspirational goal. Absent that, sustained suppression through vaccination, vigilant surveillance, and public cooperation is the realistic imperative.

In practical terms, MENA health ministries should inventory existing smallpox vaccine reserves and assess cold-chain integrity, negotiate advance purchase agreements for third-generation vaccines, incorporate mpox into notifiable-disease statutes with clear case definitions, integrate mpox PCR targets into multiplex febrile-rash diagnostic panels, and establish regional training hubs for biosafety and outbreak analytics. None of these actions requires novel technology; each demands political will, inter-sectoral coordination, and a modest financial commitment compared with the social and economic toll of an uncontrolled epidemic.

Time is unlikely to afford a generous rehearsal. Whether sparked by a zoonotic leap or a laboratory accident, the next mpox flare could reach urban centers in days. The MENA region, endowed with a youthful demographic and strategic crossroads geography, can choose preparedness over reaction. The WHO can aid in facilitating efforts for the MENA region that synthesize tracking, prevention, and coordination across different health ministries. Furthermore, multilateral donors can pool money for targeted responses to emerging crises and work towards emphasizing further research and robust documentation efforts. By weaving together lessons from previous outbreaks, leveraging health systems to personalize immunization, and confronting vaccine hesitancy head-on, policymakers can transform potential vulnerability into a showcase of resilient public health. The virological dice will always roll, but the readiness of our systems determines whether an outbreak becomes a footnote or a crisis. 
